# Book Review: Positive Psychology in Second and Foreign Language Education

**DOI:** 10.3389/fpsyg.2021.716932

**Published:** 2021-07-08

**Authors:** Zhiwu Zhang, Jinfen Xu

**Affiliations:** ^1^School of Foreign Languages, Huazhong University of Science and Technology, Wuhan, China; ^2^School of Foreign Studies, Changsha University of Science and Technology, Changsha, China

**Keywords:** positive psychology, positive emotions, second language education, foreign language education, positive language education, second language acquisition

Positive Psychology proclaimed its arrival and revival at the turn of the new millennium with the publication of Seligman and Csikszentmihalyi's 2000 seminal paper. This new branch of psychology highlights positive aspects of human experience instead of what goes wrong in life. Scholarly interest in positive psychology in Second Language Acquisition (SLA) (Gabryś-Barker and Gałajda, 2016; MacIntyre et al., 2019) has shifted from an exclusive focus on negative emotional dimension in applied linguistics (Dewaele et al., 2019) to positive emotions, such as enjoyment, self-efficacy, and confidence, thus leading to a reorientation of what we can achieve from creating positive emotions, rather than what remedies we can resort to. The edited volume *Positive Psychology in Second and Foreign Language Education*, with its new findings in such topics as well-being, enjoyment and empathy, and under-explored ones, including Positive Language Education and positive institutional policies in language education settings, presents a timely addition to the emerging field of positive psychology in second and foreign language education.

This volume consists of three parts: theoretical (Chapters 1–4), empirical (Chapters 5–9) and applied (Chapters 10–11). In Chapter 1, Peter D. MacIntyre presents an overview of positive psychology by explicating what it is and what it is not, together with a sketch of its origins and development, highlighting its applications and implications in theory, research and practice. Chapter 2 explores foreign language teacher well-being from an ecological perspective, and proposes the construct of social-psychological capital to outline resources and affordances that teachers could make use of. It is indicated that to help teachers flourish, their needs, strengths, and resources must be fully understood. Chapter 3 addresses educators' roles in creating an emotionally responsive learning environment in international higher education, centering around building positive teacher-student and student-student relationships. Chapter 4 investigates gender differences in foreign language enjoyment (FLE) through a close examination of empirical studies in this field.

The five empirical studies in Part Two discuss such topics as motivation, willingness to communicate, engagement, Positive Language Education and teacher identity in various educational settings. Chapter 5 examines the stability of positive self, positive L2 self and motivational L2 self-efficacy of Japanese college students based on the three levels of self: a global level, a domain level and a situational level. Chapter 6 investigates, by drawing on data from Polish junior English majors, the link between students' willingness to communicate, their classroom engagement and communicative behavior quantified as turn-taking and word count. In Chapter 7, Katarzyna Budzińska expounds on a new notion of Positive Language Education (PLE) from Seligman's PERMA model, and examines institutional policies that embody and enact PLE in a Polish private language school. Ten features contributing to PLE are listed, and it is suggested that the construction of positive institutions should take a more comprehensive examination in their policies, structures, and organizational cultures. Chapter 8 explores the emergence of teacher identities through positive psychology-related experiences from a longitudinal duoethnographic study of pre-service English teachers. Due to the intricate interplay between affiliation, attachment, and autonomy, pre-service teacher identity is undergoing constant emergence and change. It is proposed that it could best be interpreted as a product and a process. Chapter 9 explores through a metaphor study how English majors in a Polish private university perceive teachers, and identifies effective components of a teacher training program that contribute to a sustainable and successful teaching career.

In Part Three, Chapter 10 investigates the application of an introductory positive psychology course in a teacher training program, with a focus on its objectives, syllabus, procedures as well as trainee teachers' reflections about the course. Results suggest that through such a course, trainees are benefited in approaching their future career in a new way, and in understanding positive relationships in personal lives. Chapter 11 presents the practice of applying biographical narrative and a life path metaphor in an adult education project, and finds that through combination of actions and reflections, participants are enabled to distinguish important goal-achieving activities in life.

However, an edited volume addressing a fledgling field in full expansion is not without pities. Firstly, most studies in the book focus on perspectives from students and teacher trainees in pre-service training programs, ignoring views and insights from in-service teachers and professionals in second and foreign language education. Besides, data collected in the empirical studies are mainly through qualitative methods, such as semi-structured interviews, narratives and duoethnography. Even in studies where questionnaires are adopted, they are generally limited to descriptive analysis.

It is almost an impossible mission to present an encompassing overview of this emerging field. Despite the imperfections stated above, this book, through its collection of theoretical, empirical and applied studies, provides insights and perspectives for both research and education practice. Thus, it is a good recommendation for students, teachers, teacher trainers and researchers, who are interested in the quest for well-being and happiness of both learners and teachers in second and foreign language education settings.

## Author Contributions

ZZ selected the book and drafted the review. JX helped select the book and provided support in framework, analysis and revision. All authors have approved it for publication.

## Conflict of Interest

The authors declare that the research was conducted in the absence of any commercial or financial relationships that could be construed as a potential conflict of interest.
